# Circulating C-X-C Motif Ligand 13 as a Biomarker for Early Predicting Efficacy of Subcutaneous Immunotherapy in Children With Chronic Allergic Rhinitis

**DOI:** 10.3389/fped.2022.872152

**Published:** 2022-05-04

**Authors:** Shenghao Cheng, Sihui Wen, Shaobing Xie, Caixia Zhang, Hua Zhang, Kelei Gao, Ruohao Fan, Zhihai Xie, Weihong Jiang

**Affiliations:** ^1^Department of Otolaryngology-Head and Neck Surgery, Xiangya Hospital of Central South University, Changsha, China; ^2^Hunan Province Key Laboratory of Otolaryngology Critical Diseases, Changsha, China; ^3^National Clinical Research Center for Geriatric Disorders, Changsha, China

**Keywords:** allergic rhinitis, subcutaneous immunotherapy, CXCL13, BAFF, children

## Abstract

**Background:**

C-X-C motif ligand 13 (CXCL13) and B cell-activating factor (BAFF) are proven to be involved in inflammatory diseases, but their role in allergic rhinitis (AR) remains unclear. The aim of this study was to investigate the role of serum CXCL13 and BAFF in AR and their clinical values as objective biomarkers to predict the efficacy of subcutaneous immunotherapy (SCIT).

**Methods:**

We prospectively recruited 90 children with AR treated with SCIT and collected their serum specimens before SCIT. One-year follow-up was conducted for all patients, and they were categorized into effective and ineffective groups based on efficacy. The serum concentrations of CXCL13 and BAFF were detected and compared between the two groups. A validation cohort of 52 responders and 26 non-responders were further assessed for both cytokines and serum CXCL13 and BAFF levels were assayed by enzyme-linked immunosorbent assay (ELISA).

**Results:**

Eighty children completed the follow-up schedule, and 56 children were categorized into the effective group and 24 children into the ineffective group. The serum levels of CXCL13 in the effective group were clearly higher than those in the ineffective group (*P* < 0.05). Receiver operating characteristic (ROC) curves revealed the potential values of CXCL13 as a biomarker in predicting the response of SCIT. Further, in the validation cohort, ELISA results demonstrated that serum CXCL13 levels were increased in responders than non-responders (*P* < 0.05). ROC curves showed good accuracy of serum CXCL13 in predicting the efficacy of SCIT.

**Conclusion:**

Our discovery–validation study demonstrated that circulating CXCL13 might serve as a novel biomarker to predict the outcome of SCIT in childhood AR. The findings indicated that CXCL13 was involved in the pathological mechanisms of AR and made help to the fundamental therapeutic mechanism of SCIT.

## Introduction

Allergic rhinitis (AR) is characterized by the symptoms of nasal itching, nasal congestion, rhinorrhea, and sneezing, which is a type I allergic disease mediated by immunoglobulin E (IgE) (1, 2). Based on recent epidemiological surveys, AR affects 10–40% of adults (3) and 2–25% of children (4) all over the world, and the prevalence increased progressively (5). As a chronic disease, AR seriously influences the patients’ health and quality of life (6), especially for children. The impact of AR is more severe as the immune system of children is not mature, and pediatric AR is prone to co-existing allergic asthma and allergic conjunctivitis (7). The pathophysiology of AR is currently not clear, the production of allergen-specific IgE antibodies by B lymphocytes has been demonstrated to be the vital component (8). Allergen-specific immunotherapy (AIT) is the only treatment that induces allergen tolerance, which has been shown to improve nasal and ocular symptoms and reduce medication consumption (9). AIT is correlated with the induction of allergen-specific IgG antibodies, especially specific IgG4 (sIgG4) antibodies. In previous studies, IgG was considered to competitively bind allergens with sIgE, inhibiting the formation of allergen IgE complexes (10) and degranulation of mast cells and basophil (11, 12). Currently, AIT could be grouped into two modalities based on the mode of administration, including subcutaneous immunotherapy (SCIT) and sublingual immunotherapy (SLIT). However, increasing evidence showed that SCIT was more effective than SLIT in reducing allergic symptoms, especially in pediatric AR (13–15). Although SCIT is more effective and compliant, this treatment is not successful for every child who has AR, and the outcome fluctuates among the different users (9, 16). Besides, the treatment process of SCIT takes over 3 years, but a reliable method or biomarker to predict the treatment response is absent. Therefore, it is of great clinical significance to identify objective indicators or strategies to predict the efficacy before onsetting SCIT.

C-X-C motif ligand 13 (CXCL13), a chemokine for B cell, has been linked to multiple biological processes, including protective antibody production in autoimmune disease, and is reported as a biomarker in many inflammatory conditions such as multiple sclerosis (MS), systemic lupus erythematosus (SLE), rheumatoid arthritis (RA), and asthma (17–20). Additionally, Patadia et al. (21) found the mRNA and protein levels of CXCL13 in patients with chronic rhinosinusitis with nasal polyp (CRSwNP) were higher than healthy controls. Meanwhile, Plager et al. (22) demonstrated by exon array that the transcription of CXCL13 was increased in asthma patients with CRSwNP and atopic dermatitis. B cell-activating factor (BAFF), also known as tumor necrosis factor superfamily member 13b, is mainly secreted by monocytes and neutrophils, which is crucial for B cell activation, differentiation, and survival. Previous publications showed that soluble BAFF levels were significantly higher in patients with MS or SLE than in controls. In parallel, the BAFF antibody belimumab for SLE treatment has been successfully developed (23). However, few studies have focused on exploring the role of these two cytokines in AR and evaluating their clinical values as predictors of SCIT response, particularly in children with chronic AR.

**FIGURE 1:**
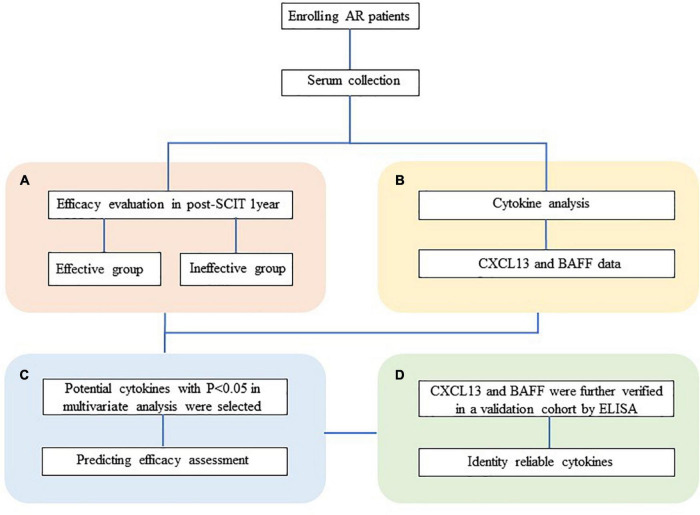
Overview of studies exploring serum predictive biomarkers of SCIT efficacy in patients with HDM-induced AR. **(A)** Follow-up and efficacy assessment. **(B)** The analysis of CXCL13 and BAFF levels in serum was performed by ELISA. **(C)** Cytokine levels were compared between the effective and ineffective groups, and their predictive ability was assessed. **(D)** Serum CXCL13 and BAFF are further validated by ELISA in a validation cohort. HDM, house dust mite; AR, allergic rhinitis; SCIT, subcutaneous immunotherapy; ELISA, enzyme-linked immunosorbent assay; CXCL13, C-X-C motif ligand 13; BAFF, B cell-activating factor.

Hence, the aim of this study was to investigate whether the serum level of CXCL13 and BAFF can be used as a biomarker to predict the efficacy of SCIT in pediatric with chronic AR patients at an early stage and then further verify the results in a validation cohort. An overview of this study profile was showed in Figure 1.

## Materials and Methods

### Participants and Settings

This study prospectively recruited 90 children with AR treated with SCIT in our medical center from July 2019 to November 2019. AR was diagnosed according to the diagnostic criteria in the guidelines for allergic rhinitis and its impact on asthma (ARIA) (3). Inclusion criteria included the following: (1) 6 ≤ age ≤ 14 years; (2) positive skin test results (at least++) for *Dermatophagoides farina* (*Der f*) and/or *Dermatophagoides pteronyssinus* (*Der p*) and/or sIgE level >0.35 IU/ml for *Der f* or *Der p*; (3) persistent AR. These subjects are those who have experienced no significant improvement in clinical symptoms after standardized pharmacological treatment and who then voluntarily choose SCIT. They were all consecutive patients treated in our center. Participants with the following conditions were excluded: having other nasal or sinus diseases, such as vasomotor rhinitis; having other inflammatory diseases, or autoimmune diseases; having active asthma; having a history of immunotherapy; having received antibiotics, corticosteroids or anti-allergic drugs within 4 weeks before the study. We collected demographic and clinical information on subjects and collected serum samples before SCIT. The exclusion criteria were used before blood sampling. For patients with dust mite and/or house dust mite (HDM) allergy who are serum specific IgE positive, we preferred to treat them with scheduled medications according to guidelines. If medication is not effective, or if the patient requests desensitization, we will consider SCIT as a next step. The baseline Visual Analog Scale (VAS) and total nasal symptom scores (TNSSs) were used to assess the severity of disease in AR patients. This was performed as follows: the VAS ranges from 0 to 10 cm, with a score of 0 corresponding to no symptoms and 10 corresponding to the worst symptoms. Each symptom category (including nasal symptoms, nasal leakage, obstruction, sneezing, and nasal itching) is rated from 0 to 10 (24). The TNSS assesses four aspects of symptom severity, including sneezing, runny nose, nasal itching and nasal obstruction. Each aspect was rated from 0 (no symptoms) to 3 (intolerably severe symptoms that interfere with daily activities). The total TNSS score is the sum of all four symptoms, with a maximum score of 12 (25).

### Measurement of Serum C-X-C Motif Ligand 13 and B Cell-Activating Factor

We collected 5 ml of fresh venous blood from all participants and stored the collected blood samples at room temperature for 1 h. Blood samples were then centrifuged at 3000 × *g* for 10 min at 4°C to obtain supernatants and stored at −80°C for subsequent experiments. Thawed and centrifuged serum samples before use. According to the manufacturer’s instructions, serum CXCL13 and BAFF levels were measured by enzyme-linked immunosorbent assay (ELISA) kit commercial (Multisciences, Hangzhou, China, 70-EK1105-96). All samples were taken in duplicate to improve testing accuracy.

### Immunotherapy

**FIGURE 2:**
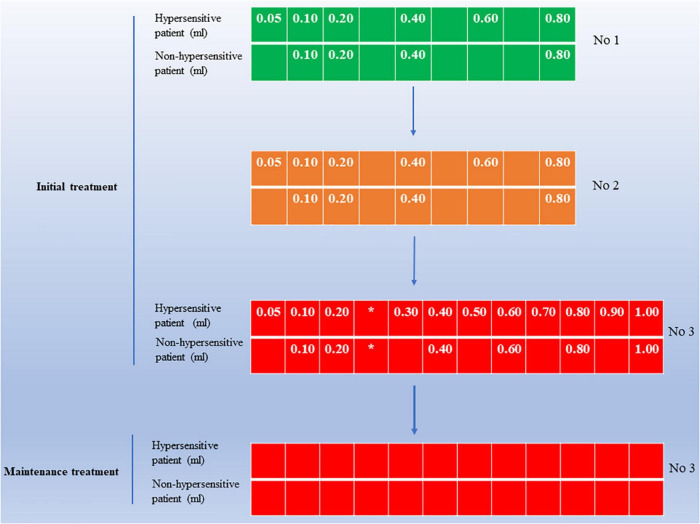
An overview of SCIT administration schedule in pediatric AR patients. The initial treatment size is available in three vials, including concentrations 1, 2, and 3. Initial treatment should start with the lowest dose at the lowest concentration 1 until the patient’s tolerance limit (individual maximum dose) is reached, with an injection interval of 7–14 days during this period. After reaching the maximum dose in the initial phase enter the maintenance phase of treatment, during which the injected dose is the individual maximum dose, with an injection interval of 4–6 weeks. For hypersensitive patients, use the dose shown in the figure for “hypersensitive patients.” *Note the maximum dose for the individual. Maximum dose concentration 3, 1.0 ml; AR, allergic rhinitis; SCIT, subcutaneous immunotherapy.

Subcutaneous immunotherapy was conducted as described previously. All pediatric AR patients received allergen extracts of *Der p* and *Der f* from Novo-Helisen-Depot (NHD) in a 1:1 ratio (Allergopharma, Reinbek, Germany). By the manufacturer’s instructions, SCIT includes two stages: initial treatment and maintenance treatment. The whole course of SCIT was recommended to last for 3–5 years to achieve long-term clinical benefits. The specific administration schedule of SCIT is shown in Figure 2.

### Clinical Outcome Assessment

We followed all patients for at least 1 year and recorded their symptoms and medication use throughout treatment. As described in previous studies, the clinical efficacy of SCIT was assessed based on improvement in clinical symptoms and reduction in medication consumption (26, 27). In brief, the nasal symptom and medication score (SMS) was defined as the sum of the TNSS and the final medication score. SMS reduction of at least 30% compared with baseline was considered to be effective; otherwise, SCIT was deemed to be ineffective.

### Validation Cohort Recruitment and Cytokine Validation

At the same time, we recruited 78 AR children treated with SCIT for more than 1 year, forming another independent cohort to confirm further the discovery of potential biomarkers in the discovery cohort. After 1 year of treatment, the early efficacy evaluation showed that 52 cases were effective and 26 cases were ineffective. Serum samples were collected from all patients to measure CXCL13 and BAFF levels using the same commercial ELISA kit (Multisciences, Hangzhou, China, 70-EK1105-96) following the manufacturer’s instructions.

### Statistical Analysis

Numerical variables were expressed as median and interquartile range. For variables with normal distribution, Student’s *t*-test was conducted. Otherwise, Mann–Whitney *U*-test was performed. Frequencies and percentages were used to describe categorical data, and Chi-square tests were applied to compare the differences. The predictive efficacy value of serum CXCL13 and BAFF was assessed using logistic regression analysis and receiver operating characteristic (ROC) curves. SPSS statistical software version 26.0 (IBM, Chicago, IL, United States) was utilized for all statistical analyses. In all tests, *P* < 0.05 was deemed statistically significant.

## Results

### Baseline Characteristics of Study Participants

A total of 80 patients in this study completed the 1-year follow-up program and provided full information. Of these had been included, 56 were categorized as the effective group, and 24 patients were enrolled in the ineffective group. The distribution of clinical variables for all subjects is summarized in Table 1. Statistical differences were not found in gender, age, body mass index (BMI), multiple allergies, concomitant diseases rate, baseline VAS, and TNSSs (all *P* > 0.05). In validation cohort, statistical differences were not found in the distribution of clinical variables for all patients in Table 2 (all *P* > 0.05).

**TABLE 1:** Demographics and clinical characteristics of patients in discovery cohort.

Variables	Effective group (*n* = 56)	Ineffective group (*n* = 24)	*P*-value
**Sex**			0.961
Male	33 (58.9%)	14 (58.3%)	
Female	23 (41.1%)	10 (41.7%)	
Age, years	10.0 (4.0)	9.7 ± 2.3	0.996
BMI, kg/m^2^	17.3 (4.1)	18.3 ± 3.1	0.698
**Multiple allergies**			0.345
Yes	7 (12.5%)	5 (20.8%)	
No	49 (87.5%)	19 (79.2%)	
**Concomitant diseases**			
Allergic asthma	15 (26.8%)	8 (33.3%)	0.559
Allergic conjunctivitis	10 (17.9%)	7 (29.2%)	0.301
Baseline VAS	6.0 (3.0)	5.9 ± 1.6	0.704
Baseline TNSS	8.0 (2.0)	8.3 ± 1.6	0.540

*BMI, body mass index; TNSS, total nasal symptom score; VAS, Visual Analog Scale.*

**TABLE 2:** Demographics and clinical characteristics of patients in validation cohort.

Variables	Effective group (*n* = 52)	Ineffective group (*n* = 26)	*P*-value
**Sex**			0.627
Male	31 (59.6%)	14 (53.8%)	
Female	21 (40.4%)	12 (46.2%)	
Age, years	10.0 (4.0)	10.4 ± 2.4	0.919
BMI, kg/m^2^	17.9 ± 4.6	17.7 (4.0)	0.687
**Multiple allergies**			0.357
Yes	6 (11.5%)	5 (19.2%)	
No	46 (88.5%)	21 (80.8%)	
**Concomitant diseases**			
Allergic asthma	15	7	0.859
Allergic conjunctivitis	10	5	1.000
Baseline VAS	6.0 (3.0)	5.0 (2.0)	0.837
Baseline TNSS	8.0 (2.0)	7.8 ± 1.4	0.738

### Changes of Serum C-X-C Motif Ligand 13 and B Cell-Activating Factor and Associated With Subcutaneous Immunotherapy Efficacy

**FIGURE 3:**
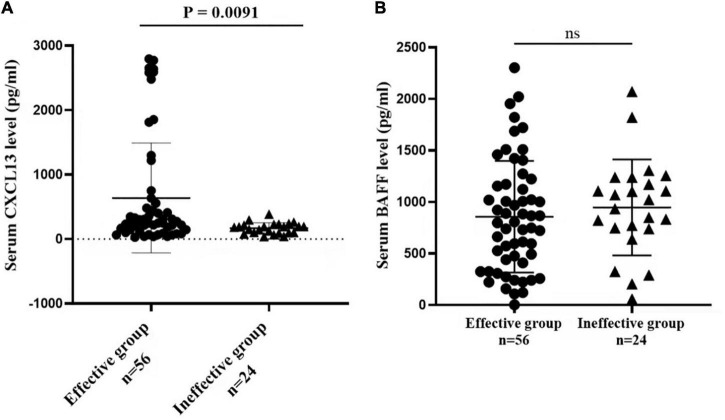
Serum cytokines levels of CXCL13 and BAFF between the effective and ineffective groups. **(A)** The serum CXCL13 levels are significantly up-regulated in the effective group than the ineffective group (*P* < 0.01). **(B)** No statistical difference is observed in serum BAFF levels between the two groups (*P* > 0.05). CXCL13, C-X-C motif ligand 13; BAFF, B cell-activating factor; AR, allergic rhinitis; SCIT, subcutaneous immunotherapy.

**FIGURE 4:**
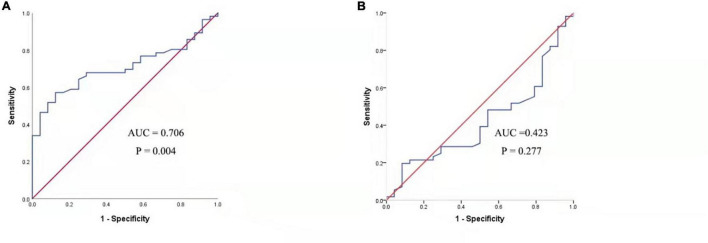
ROC analysis of serum CXCL13 level **(A)** and BAFF level **(B)** in predicting the clinical outcome of SCIT in pediatric AR patients. CXCL13, C-X-C motif ligand 13; SCIT, subcutaneous immunotherapy; ROC, receiver operating characteristics; AUC, area under the curve; AR, allergic rhinitis; BAFF, B cell-activating factor.

Figure 3 presented that the peripheral concentrations of CXCL13 were increased in the effective group compared to the ineffective group (*P* < 0.05), but the serum levels of BAFF were not statistically different between the two groups (*P* > 0.05). Unadjusted and adjusted multivariate analysis results showed that circulating levels of CXCL13 are strongly linked to the efficacy in pediatric AR with SCIT (Table 3). ROC curves result showed that the circulating CXCL13 area under the curve (AUC = 0.706, *P* = 0.004) showed potential application in the prediction of the efficacy of SCIT (Figure 4). Detailed parameters were presented in Table 4.

**TABLE 3:** Binary logistic regression exploring factors associated with SCIT efficacy.

Variables	Unadjusted		Adjusted	
		
	OR (95% CI)	*P*-value	OR (95% CI)	*P*-value
Sex	0.976 (0.370–2.575)	0.960	0.781 (0.223–2.740)	0.699
Age, years	0.994 (0.808–1.223)	0.958	1.010 (0.795–1.284)	0.932
BMI, kg/m^2^	1.022 (0.883–1.183)	0.770	1.041 (0.875–1.238)	0.653
Multiple allergies	0.543 (0.153–1.921)	0.343	0.536 (0.112–2.564)	0.435
Allergic asthma	0.732 (0.260–2.059)	0.554	0.692 (0.210–2.279)	0.545
Allergic conjunctivitis	0.528 (0.173–1.609)	0.261	0.832 (0.236–2.931)	0.775
Baseline VAS	1.076 (0.802–1.444)	0.624	1.381 (0.602–3.167)	0.446
Baseline TNSS	1.112 (0.830–1.490)	0.477	0.793 (0.350–1.797)	0.579
CXCL13	0.996 (0.991–1.000)	0.039	0.995 (0.990–1.000)	0.031
BAFF	1.000 (0.999–1.001)	0.472	1.000 (0.999–1.001)	0.564

**TABLE 4:** Receiver operating characteristic analysis results of different predictors for SCIT efficacy.

Variables	AUC (95% CI)	*P*-value	Cutoff value	Sensitivity	Specificity
CXCL-13	0.706 (0.596–0.817)	0.004	244.2554	0.571	0.875
BAFF	0.423 (0.290–0.556)	0.277	1355.266	0.196	0.917

### Serum Levels of C-X-C Motif Ligand 13 and B Cell-Activating Factor in the Validation Cohort

**FIGURE 5:**
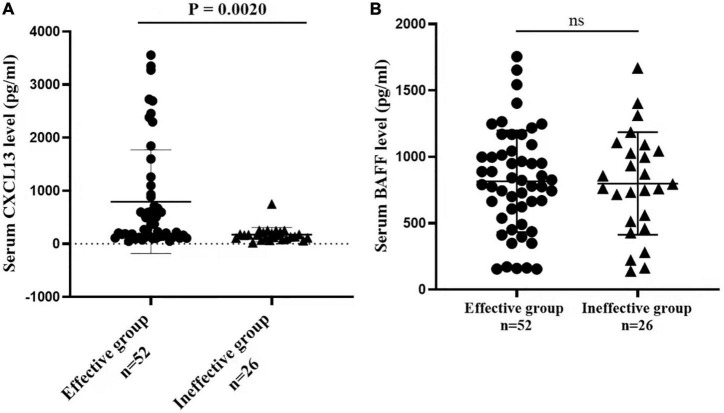
The serum levels of **(A)** CXCL13 **(B)** BAFF between valid and invalid groups in the validation queue. The CXCL13 level is significantly increased in the effective group in comparison with the ineffective group (*P* < 0.01). The BAFF level is no statistical difference between the two groups (*P* > 0.05). CXCL13, C-X-C motif ligand 13; BAFF, B cell-activating factor.

**FIGURE 6:**
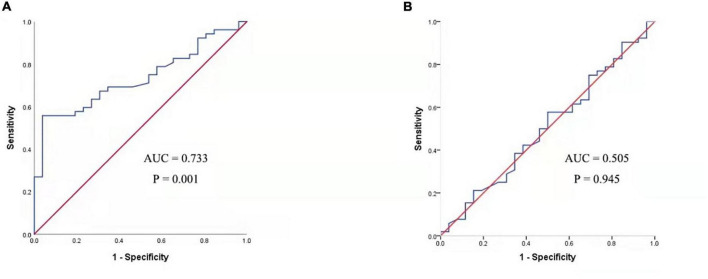
ROC analysis of serum CXCL13 level **(A)** and BAFF level **(B)** in predicting the clinical outcome of SCIT in a validation cohort. CXCL13, C-X-C motif ligand 13; SCIT, subcutaneous immunotherapy; ROC, receiver operating characteristics; AUC, area under the curve; AR, allergic rhinitis; BAFF, B cell-activating factor.

The serum concentrations of CXCL13 and BAFF were detected in a validation cohort of 52 responders and 26 non-responders who completed 1 year of SCIT for further confirmation of the findings above. In terms of demographic and clinical data, no statistical differences were observed between the two groups. Compared to the invalid group, serum CXCL13 levels were markedly higher in the valid group (*P* < 0.05, Figure 5). Serum BAFF expression levels in both groups were not statistically different. Besides, binary logistic regression analysis showed that CXCL13 expression levels were intimately related to the efficacy of SCIT in the validation cohort (Table 5). The ROC curve indicated that serum CXCL13 (AUC = 0.733, *P* = 0.001) displayed a promising application in predicting the outcome of SCIT in Figure 6, and the detailed parameters were shown in Table 6.

**TABLE 5:** Binary logistic regression exploring factors associated with SCIT efficacy in validation cohort.

Variables	Unadjusted		Adjusted	
		
	OR (95% CI)	*P*-value	OR (95% CI)	*P*-value
Sex	0.790 (0.306–2.042)	0.627	0.720 (0.229–2.259)	0.573
Age, years	1.003 (0.829–1.214)	0.974	1.024 (0.808–1.296)	0.846
BMI, kg/m^2^	1.016 (0.905–1.140)	0.791	1.123 (0.949–1.330)	0.178
Multiple allergies	1.825 (0.500–6.659)	0.362	0.467 (0.092–2.379)	0.360
Allergic asthma	1.100 (0.384–3.157)	0.859	0.905 (0.244–3.355)	0.882
Allergic conjunctivitis	1.000 (0.303–3.302)	1.000	0.858 (0.211–3.489)	0.831
Baseline VAS	0.971 (0.725–1.300	0.842	1.410 (0.538–3.697)	0.485
Baseline TNSS	0.957 (0.716–1.280)	0.767	0.810 (0.0.316–2.076)	0.660
CXCL13	0.996 (0.992–0.999)	0.013	0.995 (0.992–0.999)	0.008
BAFF	1.000 (0.999–1.001)	0.857	0.999 (0.998–1.001)	0.329

**TABLE 6:** Receiver operating characteristic analysis results of different predictors for SCIT efficacy in validation cohort.

Variables	AUC (95% CI)	*P*-value	Cutoff value	Sensitivity	Specificity
CXCL-13	0.733 (0.623–0.842)	0.001	261.9004	0.558	0.962
BAFF	0.505 (0.368–0.642)	0.945	769.0156	0.557	0.500

## Discussion

Previous studies have shown that in southern China, HDM was the most widespread allergen in patients with AR (28). The SCIT was recommended as the preferred treatment for AR patients caused by HDM, especially in pediatric with chronic AR, and its efficacy has been proved and widely used in clinical practice (13, 29, 30). Although SCIT can effectively alleviate clinical symptoms and improve the quality of life for AR patients, only certain subsets of patients benefit from this therapy (31, 32). Therefore, exploring an appropriate method or biomarker is urgently needed to predict SCIT efficacy, which contributes to early screening out those patients who are most likely to benefit from SCIT. While some parameters were thought to be possible indicators for predicting the efficacy of SCIT, such as T cell modulation/regulatory T cells (Tregs) appearance (33), specific IgE/total-IgE ratio (34), their predictive value was still under debate. To address this issue, we conducted a discover–validation study to explore potential biomarkers that could be used to predict the efficacy of SCIT in pediatric with chronic AR patients. In this study, these results demonstrated that serum CXCL13 expression levels were up-regulated in the effective group compared with the ineffective group, and displayed reliable accuracy in the prediction of the SCIT efficacy. Moreover, the predictive ability of CXCL13 was confirmed in a validation cohort. The above results indicated that circulating CXCL13 was participating in the response to SCIT and its potential therapeutic mechanisms.

Previous publications have highlighted that B cells were involved in the pathological of AR through differentiation into plasma cells and secreted specific IgE (8, 35). During AIT, conversion of allergen-specific IgE B cells to isotypes that produce blocking antibodies with the same antigenic specificity (36, 37). CXCL13, as B cell chemokine, can attract B cells into the lesion tissue and produce specific antibodies, such as IgG4 and IgA, when AIT is responsive (38). Moreover, an increase in serum levels of IgG and IgG4 antibodies against allergens has been a crucial indicator regarded in successful AIT (39–41). Interestingly, a classic study found that beekeepers were frequently exposed to bee venom, the number of regulatory B cells (Bregs) were increased and the IgG4 production by the Bregs was enhanced (42). Furthermore, a prior study confirmed that Bregs had been shown to suppress Th2 cells and induce Treg cells, then inhibited the Th2 allergic inflammatory responses (43). As is well known, Th2 inflammation dominates the pathogenesis of AR and underlies the mechanism of SCIT treatment (30, 32). Immunotherapy has been proved to modulate the balance of Th1/Th2 responses, and up-regulation of Th1, regulatory B, and T cells were thought to improve prognosis (44). In this study, we demonstrated that the levels of serum CXCL13 were higher in the effective group than the ineffective group, and circulating CXCL13 before SCIT was positively correlated with the efficacy of SCIT. These findings imply that serum CXCL13 may be a reliable biomarker for the prediction of SCIT efficacy and is involved in the therapeutic mechanism of SCLT. Lee et al. found that the mean CXCL13 levels were markedly greater in patients suffering from lupus nephritis or in the presence of autoantibodies, suggesting that CXCL13 may be involved in the pathophysiological processes of lupus nephritis (18). Similarly, Al-Kufaidy et al. demonstrated that enhancement of B-cell migration by IL-17 during asthma induces CXCL13 chemokine secretion that is present in structural lung cells (45). Given that, we were reasonably confident that increased levels of circulating CXCL13 facilitated the evolution of immune tolerance in the period of SCIT.

The present study has some limitations. Firstly, this study was confined to a relatively small sample with only one medical center, which might increase the potential hazard of selection bias. Secondly, the standards for assessing the efficacy of SCIT have not been developed by international consensus. Finally, this might undermine the conclusions given the rather limited follow-up period. Continuing to monitor all participants and conducting further studies to confirm our findings is our next step.

In summary, this study is the first discovered–validation study to explore the potential role of CXCL13 and BAFF in pediatric with chronic AR patients and their clinical values as objective biomarkers for predicting the efficacy of SCIT. Our results revealed that pediatric AR patients who responded to SCIT were characterized by higher baseline circulating CXCL13 levels. Thus, CXCL13 may potentially be used as a biomarker to predict the efficacy of SCIT in Chinese children with HDM-induced AR. In addition, the research further reinforces the proof that serum chemokines are closely related to SCIT response and facilitates the understanding of potential mechanisms of treatment.

## Data Availability Statement

The raw data supporting the conclusions of this article will be made available by the authors, without undue reservation.

## Ethics Statement

The studies involving human participants were reviewed and approved by the Human Ethical Committee of Xiangya Hospital of Central South University. Written informed consent to participate in this study was provided by the participants’ legal guardian/next of kin.

## Author Contributions

ZX and WJ designed the study. SW and SC wrote the manuscript. SX, CZ, and HZ collected the sample and performed the data analysis. KG and RF provided the statistical support. All authors contributed to the article and approved the submitted version.

## Conflict of Interest

The authors declare that the research was conducted in the absence of any commercial or financial relationships that could be construed as a potential conflict of interest.

## Publisher’s Note

All claims expressed in this article are solely those of the authors and do not necessarily represent those of their affiliated organizations, or those of the publisher, the editors and the reviewers. Any product that may be evaluated in this article, or claim that may be made by its manufacturer, is not guaranteed or endorsed by the publisher.

## References

[1] Chen X Xie ZH Lv YX Tang QP Zhang H Zhang JY A proteomics analysis reveals that A2M might be regulated by STAT3 in persistent allergic rhinitis. *Clin Exp Allergy.* (2016) 46:813–24. 10.1111/cea.12711 27228572

[2] Xie S Zhang H Wang F Liu Y Gao K Zhang J Activated leukocyte cell adhesion molecule as a biomarker for disease severity and efficacy of sublingual immunotherapy in allergic rhinitis. *Int Immunopharmacol.* (2020) 88:106975. 10.1016/j.intimp.2020.106975 33182046

[3] Brozek JL Bousquet J Agache I Agarwal A Bachert C Bosnic-Anticevich S Allergic rhinitis and its impact on asthma (ARIA) guidelines-2016 revision. *J Allergy Clin Immunol.* (2017) 140:950–8. 10.1016/j.jaci.2017.03.050 28602936

[4] Zhang Y Zhang L. Increasing prevalence of allergic rhinitis in China. *Allergy Asthma Immunol Res.* (2019) 11:156–69. 10.4168/aair.2019.11.2.156 30661309 PMC6340797

[5] Zhang Y Lan F Zhang L. Advances and highlights in allergic rhinitis. *Allergy.* (2021) 76:3383–9. 10.1111/all.15044 34379805

[6] Bousquet J Hellings PW Agache I Amat F Annesi-Maesano I Ansotegui IJ Allergic rhinitis and its impact on asthma (ARIA) Phase 4 (2018): change management in allergic rhinitis and asthma multimorbidity using mobile technology. J Allergy Clin Immunol. 2019 Mar; 143(3): 864-879. *Erratum J Allergy Clin Immunol.* (2019) 144:1456. 10.1016/j.jaci.2018.08.049 30273709

[7] Schuler Iv CF Montejo JM. Allergic rhinitis in children and adolescents. *Pediatr Clin North Am.* (2019) 66:981–93. 10.1016/j.pcl.2019.06.004 31466686

[8] Eifan AO Durham SR. Pathogenesis of rhinitis. *Clin Exp Allergy.* (2016) 46:1139–51. 10.1111/cea.12780 27434218

[9] Liu Z Lu H Feng X Hu L Wang J Yu H. Predictive methods for efficacy of house dust mite subcutaneous immunotherapy in allergic rhinitis patients: a prospective study in a Chinese population. *Int Forum Allergy Rhinol.* (2020) 10:314–9. 10.1002/alr.22508 31869861

[10] Aalberse R. The role of IgG antibodies in allergy and immunotherapy. *Allergy.* (2011) 66 (Suppl. 95):28–30. 10.1111/j.1398-9995.2011.02628.x 21668848

[11] Frew AJ. Allergen immunotherapy. *J Allergy Clin Immunol.* (2010) 125 (2 Suppl. 2):S306–13. 10.1016/j.jaci.2009.10.064 20176266

[12] van de Veen W Stanic B Wirz OF Jansen K Globinska A Akdis M. Role of regulatory B cells in immune tolerance to allergens and beyond. *J Allergy Clin Immunol.* (2016) 138:654–65. 10.1016/j.jaci.2016.07.006 27596706

[13] Dretzke J Meadows A Novielli N Huissoon A Fry-Smith A Meads C. Subcutaneous and sublingual immunotherapy for seasonal allergic rhinitis: a systematic review and indirect comparison. *J Allergy Clin Immunol.* (2013) 131:1361–6. 10.1016/j.jaci.2013.02.013 23557834

[14] Durham SR Penagos M. Sublingual or subcutaneous immunotherapy for allergic rhinitis? *J Allergy Clin Immunol.* (2016) 137:339–49.e10. 10.1016/j.jaci.2015.12.1298 26853126

[15] Pfaar O Lou H Zhang Y Klimek L Zhang L. Recent developments and highlights in allergen immunotherapy. *Allergy.* (2018) 73:2274–89. 10.1111/all.13652 30372537

[16] Endaryanto A Nugraha RA. Indonesia-based study of the clinical and cost-saving benefits of subcutaneous allergen immunotherapy for children with allergic rhinitis in private practice. *Cells.* (2021) 10:1841. 10.3390/cells10071841 34360010 PMC8303991

[17] Alvarez E Piccio L Mikesell RJ Klawiter EC Parks BJ Naismith RT CXCL13 is a biomarker of inflammation in multiple sclerosis, neuromyelitis optica, and other neurological conditions. *Mult Scler.* (2013) 19:1204–8. 10.1177/1352458512473362 23322500 PMC3959125

[18] Lee HT Shiao YM Wu TH Chen WS Hsu YH Tsai SF Serum BLC/CXCL13 concentrations and renal expression of CXCL13/CXCR5 in patients with systemic lupus erythematosus and lupus nephritis. *J Rheumatol.* (2010) 37:45–52. 10.3899/jrheum.090450 19955043

[19] Rosengren S Wei N Kalunian KC Kavanaugh A Boyle DL. CXCL13: a novel biomarker of B-cell return following rituximab treatment and synovitis in patients with rheumatoid arthritis. *Rheumatology (Oxford).* (2011) 50:603–10. 10.1093/rheumatology/keq337 21098574

[20] Baay-Guzman GJ Huerta-Yepez S Vega MI Aguilar-Leon D Campillos M Blake J Role of CXCL13 in asthma: novel therapeutic target. *Chest.* (2012) 141:886–94. 10.1378/chest.11-0633 22016489

[21] Patadia M Dixon J Conley D Chandra R Peters A Suh LA Evaluation of the presence of B-cell attractant chemokines in chronic rhinosinusitis. *Am J Rhinol Allergy.* (2010) 24:11–6. 10.2500/ajra.2010.24.3386 20109310 PMC3335771

[22] Plager DA Kahl JC Asmann YW Nilson AE Pallanch JF Friedman O Gene transcription changes in asthmatic chronic rhinosinusitis with nasal polyps and comparison to those in atopic dermatitis. *PLoS One.* (2010) 5:e11450. 10.1371/journal.pone.0011450 20625511 PMC2897889

[23] Croft M Siegel RM. Beyond TNF: TNF superfamily cytokines as targets for the treatment of rheumatic diseases. *Nat Rev Rheumatol.* (2017) 13:217–33. 10.1038/nrrheum.2017.22 28275260 PMC5486401

[24] Klimek L Bergmann KC Biedermann T Bousquet J Hellings P Jung K Visual analogue scales (VAS): measuring instruments for the documentation of symptoms and therapy monitoring in cases of allergic rhinitis in everyday health care: position paper of the German society of allergology (AeDA) and the German society of allergy and clinical immunology (DGAKI), ENT section, in collaboration with the working group on clinical immunology, allergology and environmental medicine of the german society of otorhinolaryngology, head and neck surgery (DGHNOKHC). *Allergo J Int.* (2017) 26:16–24. 10.1007/s40629-016-0006-7 28217433 PMC5288410

[25] Benninger M Farrar JR Blaiss M Chipps B Ferguson B Krouse J Evaluating approved medications to treat allergic rhinitis in the United States: an evidence-based review of efficacy for nasal symptoms by class. *Ann Allergy Asthma Immunol.* (2010) 104:13–29. 10.1016/j.anai.2009.11.020 20143641

[26] Erekosima N Suarez-Cuervo C Ramanathan M Kim JM Chelladurai Y Segal JB Effectiveness of subcutaneous immunotherapy for allergic rhinoconjunctivitis and asthma: a systematic review. *Laryngoscope.* (2014) 124:616–27. 10.1002/lary.24295 23832632

[27] Tahamiler R Saritzali G Canakcioglu S Ozcora E Dirican A. Comparison of the long-term efficacy of subcutaneous and sublingual immunotherapies in perennial rhinitis. *ORL.* (2008) 70:144–50. 10.1159/000124286 18391573

[28] Li J Sun B Huang Y Lin X Zhao D Tan G A multicentre study assessing the prevalence of sensitizations in patients with asthma and/or rhinitis in China. *Allergy.* (2009) 64:1083–92. 10.1111/j.1398-9995.2009.01967.x 19210346

[29] Calderon MA Alves B Jacobson M Hurwitz B Sheikh A Durham S. Allergen injection immunotherapy for seasonal allergic rhinitis. *Cochrane Database Syst Rev.* (2007) 2007:CD001936. 10.1002/14651858.CD001936.pub2 17253469 PMC7017974

[30] Zissler UM Schmidt-Weber CB. Predicting success of allergen-specific immunotherapy. *Front Immunol.* (2020) 11:1826. 10.3389/fimmu.2020.01826 32983092 PMC7477353

[31] Gotoh M Kaminuma O Nakaya A Katayama K Motoi Y Watanabe N Identification of biomarker sets for predicting the efficacy of sublingual immunotherapy against pollen-induced allergic rhinitis. *Int Immunol.* (2017) 29:291–300. 10.1093/intimm/dxx034 28575522

[32] Celebi Sözener Z Mungan D Cevhertas L Ogulur I Akdis M Akdis C. Tolerance mechanisms in allergen immunotherapy. *Curr Opin Allergy Clin Immunol.* (2020) 20:591–601. 10.1097/ACI.0000000000000693 33002895

[33] Maggi E. T-cell responses induced by allergen-specific immunotherapy. *Clin Exp Immunol.* (2010) 161:10–8. 10.1111/j.1365-2249.2010.04148.x 20408857 PMC2940143

[34] Liu W Zeng Q Luo R. Predictors for short-term efficacy of allergen-specific sublingual immunotherapy in children with allergic rhinitis. *Mediators Inflamm.* (2020) 2020:1847061. 10.1155/2020/1847061 32377158 PMC7191440

[35] Gowthaman U Chen JS Zhang B Flynn WF Lu Y Song W Identification of a T follicular helper cell subset that drives anaphylactic IgE. *Science.* (2019) 365:eaaw6433. 10.1126/science.aaw6433 31371561 PMC6901029

[36] Groh N von Loetzen CS Subbarayal B Möbs C Vogel L Hoffmann A IgE and allergen-specific immunotherapy-induced IgG4 recognize similar epitopes of Bet v 1, the major allergen of birch pollen. *Clin Exp Allergy.* (2017) 47:693–703. 10.1111/cea.12835 27770477

[37] Akdis CA Blesken T Akdis M Wüthrich B Blaser K. Role of interleukin 10 in specific immunotherapy. *J Clin Invest.* (1998) 102:98–106. 10.1172/JCI2250 9649562 PMC509070

[38] Lawrence MG Steinke JW Borish L. Basic science for the clinician: mechanisms of sublingual and subcutaneous immunotherapy. *Ann Allergy Asthma Immunol.* (2016) 117:138–42. 10.1016/j.anai.2016.06.027 27499541 PMC4978173

[39] Shamji MH Ljørring C Francis JN Calderon MA Larché M Kimber I Functional rather than immunoreactive levels of IgG4 correlate closely with clinical response to grass pollen immunotherapy. *Allergy.* (2012) 67:217–26. 10.1111/j.1398-9995.2011.02745.x 22077562

[40] Scadding GW Shamji MH Jacobson MR Lee DI Wilson D Lima MT Sublingual grass pollen immunotherapy is associated with increases in sublingual Foxp3-expressing cells and elevated allergen-specific immunoglobulin G4, immunoglobulin A and serum inhibitory activity for immunoglobulin E-facilitated allergen binding to B cells. *Clin Exp Allergy.* (2010) 40:598–606. 10.1111/j.1365-2222.2010.03462.x 20184605

[41] Shamji MH Francis JN Würtzen PA Lund K Durham SR Till SJ. Cell-free detection of allergen-IgE cross-linking with immobilized phase CD23: inhibition by blocking antibody responses after immunotherapy. *J Allergy Clin Immunol.* (2013) 132:1003–5.e1–4. 10.1016/j.jaci.2013.05.025 23827773

[42] van de Veen W Stanic B Yaman G Wawrzyniak M Söllner S Akdis DG IgG4 production is confined to human IL-10-producing regulatory B cells that suppress antigen-specific immune responses. *J Allergy Clin Immunol.* (2013) 131:1204–12. 10.1016/j.jaci.2013.01.014 23453135

[43] Amu S Saunders SP Kronenberg M Mangan NE Atzberger A Fallon PG. Regulatory B cells prevent and reverse allergic airway inflammation via FoxP3-positive T regulatory cells in a murine model. *J Allergy Clin Immunol.* (2010) 125:1114–24.e8. 10.1016/j.jaci.2010.01.018 20304473

[44] Breiteneder H Peng YQ Agache I Diamant Z Eiwegger T Fokkens WJ Biomarkers for diagnosis and prediction of therapy responses in allergic diseases and asthma. *Allergy.* (2020) 75:3039–68. 10.1111/all.14582 32893900 PMC7756301

[45] Al-Kufaidy R Vazquez-Tello A BaHammam AS Al-Muhsen S Hamid Q Halwani R. IL-17 enhances the migration of B cells during asthma by inducing CXCL13 chemokine production in structural lung cells. *J Allergy Clin Immunol.* (2017) 139:696–9.e5. 10.1016/j.jaci.2016.07.037 27639935

